# Maternal but Not Infant Anti-HIV-1 Neutralizing Antibody Response Associates with Enhanced Transmission and Infant Morbidity

**DOI:** 10.1128/mBio.01373-17

**Published:** 2017-10-24

**Authors:** Melissa Ghulam-Smith, Alex Olson, Laura F. White, Charles S. Chasela, Sascha R. Ellington, Athena P. Kourtis, Denise J. Jamieson, Gerald Tegha, Charles M. van der Horst, Manish Sagar

**Affiliations:** a Department of Medicine, Boston University School of Medicine, Boston, Massachusetts, USA; b Department of Biostatistics, Boston University School of Public Health, Boston, Massachusetts, USA; c University of the Witwatersrand, Johannesburg, South Africa; d Division of Reproductive Health, Centers for Disease Control and Prevention, Atlanta, Georgia, USA; e University of North Carolina Project-Malawi, Lilongwe, Malawi; f University of North Carolina, Chapel Hill, North Carolina, USA; Fred Hutchinson Cancer Research Center; Johns Hopkins Bloomberg School of Public Health

**Keywords:** HIV, MTCT, antibodies, breast milk, infant mortality

## Abstract

A significant number of infants acquire HIV-1 through their infected mother’s breast milk, primarily due to limited access to antiretrovirals. Passive immunization with neutralizing antibodies (nAbs) may prevent this transmission. Previous studies, however, have generated conflicting results about the ability of nAbs to halt mother-to-child transmission (MTCT) and their impact on infant outcomes. This study compared plasma neutralizing activity in exposed infants and the infected mothers (*n =* 63) against heterologous HIV-1 variants and the quasispecies present in the mother. HIV-exposed uninfected infants (HEU) (*n =* 42), compared to those that eventually acquired infection (*n =* 21), did not possess higher nAb responses against heterologous envelopes (*P* = 0.46) or their mothers’ variants (*P* = 0.45). Transmitting compared to nontransmitting mothers, however, had significantly higher plasma neutralizing activity against heterologous envelopes (*P* = 0.03), although these two groups did not have significant differences in their ability to neutralize autologous strains (*P* = 0.39). Furthermore, infants born to mothers with greater neutralizing breadth and potency were significantly more likely to have a serious adverse event (*P* = 0.03). These results imply that preexisting anti-HIV-1 neutralizing activity does not prevent breast milk transmission. Additionally, high maternal neutralizing breadth and potency may adversely influence both the frequency of breast milk transmission and subsequent infant morbidity.

## IMPORTANCE

Passive immunization trials are under way to understand if preexisting antibodies can decrease mother-to-child HIV-1 transmission and improve infant outcomes. We examined the influence of preexisting maternal and infant neutralizing activity on transmission and infant morbidity in a breastfeeding mother-infant cohort. Neutralization was examined against both the exposure strains circulating in the infected mothers and a standardized reference panel previously used to estimate breadth. HIV-exposed uninfected infants did not possess a broader and more potent response against both the exposure and heterologous strains compared to infants that acquired infection. Transmitting, compared to nontransmitting, mothers had significantly higher neutralization breadth and potency but similar responses against autologous variants. Infants born to mothers with higher neutralization responses were more likely to have a serious adverse event. Our results suggest that preexisting antibodies do not protect against breast milk HIV-1 acquisition and may have negative consequences for the baby.

## INTRODUCTION

In the absence of intervention, approximately 60% of infants born to HIV-infected mothers will escape infection despite constant exposure to the virus *in utero*, during labor, and throughout breastfeeding (1, 2). Multiple factors, such as maternal virus level, maternal immunologic status, the presence of other infections, and human leukocyte antigen concordance, are known to influence transmission frequency (1). Antibodies acquired by the baby from the mother during gestation and breastfeeding may also prevent transmission, although previous studies have not provided a definitive answer (3). Studies have also suggested that the acquired maternal antibodies can impact infant morbidity (4–7). The goal of this work is to investigate the influence of humoral immunity on breastfeeding HIV-1 transmission and infant outcomes. A better understanding of the immune mechanisms that impact mother-to-child transmission (MTCT) and infant mortality is crucial for developing prevention strategies to eliminate new infections and improve infant health.

During the course of HIV-1 infection, host antibodies emerge against envelope (Env) glycoproteins, which are composed of surface gp120 and transmembrane gp41 subunit trimers (8). These Env spikes mediate viral entry into host cells and are the sole targets of neutralizing antibodies (nAbs) (9). Upon binding, antibodies can prevent cell-free virus from entering host target cells or obstruct cell-to-cell virus transfer (10, 11). Early in infection, the host develops antibodies that block autologous virus (9), but viruses respond by acquiring extensive Env sequence and glycosylation variation, which confers immune evasion (12, 13). Over time, some infected individuals may develop nAbs that can block heterologous viral variants by binding conserved Env epitopes, such as the CD4-binding site (CD4bs), V1/V2 or V3 loop glycans, the gp120-gp41 interface, or the gp41 membrane-proximal external region (MPER) (14, 15). The ability of these types of antibodies, termed “broadly neutralizing antibodies” (bnAbs), to recognize diverse viral strains is termed “breadth,” while “potency” describes the amount of nAb needed to inhibit a fixed virus inoculum (16). Broad and potent monoclonal antibodies (MAbs) have been generated from cells obtained from individuals with heterologous neutralization responses. Some of the isolated bnAbs are being examined in passive immunization studies to prevent MTCT (17, 18).

HIV-1 vaccine efforts are focused on immunogen design capable of eliciting these bnAbs prior to exposure (19). Although current immunogens are unable to elicit bnAbs, it has been demonstrated that passive immunization of diverse bnAbs successfully prevents virus acquisition in animal models, which justifies the efforts for vaccine development (20, 21). Animal studies, however, do not account for diverse factors present during human transmission (22). For instance, animal challenge studies are primarily conducted with a neutralization-sensitive cell-free virus and generally with nonphysiologic high levels of passively administered antibodies (23). In many ways, MTCT provides an ideal model to assess if antibodies can prevent transmission among humans because the fetus begins acquiring maternal immunoglobulins G (IgGs) as early as 13 weeks of gestation (24). The rate of acquisition increases throughout pregnancy, with the largest amount of transfer occurring in the third trimester (25). Therefore, at birth, infants are primed with amounts of IgG equal to if not greater than those of their mother (24). In the setting of HIV-1, virus-specific antibodies are efficiently transferred to infants *in utero* (26) and through breastfeeding (27). The presence of high levels of maternal nAbs in the infant peri- and postpartum represents a favorable scenario in which neutralization of incoming maternal variants may prevent infection.

The MTCT model had been studied previously to gain a better understanding of humoral immunity and prevention of mother-to-child transmission (PMTCT), but these earlier investigations often yielded conflicting conclusions. Some previous results have suggested that antibodies reduce transmission because nontransmitting mothers (NTMs) were shown to have higher neutralizing or binding titers compared to transmitting mothers (TMs) (28–30). In addition, exposed infants that eventually become infected acquire a limited number of HIV-1 variants, even though the infected mother harbors a diverse quasispecies (31). These infecting strains have been found to be neutralization resistant to maternal antibodies, further suggesting that the maternal humoral response impacts transmission (32, 33). In contrast, some investigations have shown an association between transmission and higher titer of neutralizing or binding antibodies, implying maternal responses potentially enhance MTCT (34–38). These previous studies may have yielded contrasting results because they often examined different modes of MTCT. Indeed, recent data suggest that *in utero*, peripartum, and breastfeeding transmission select for viruses with distinct characteristics with potentially unique neutralization profiles (39). Importantly, only a limited number of studies have examined infant responses (7, 40), and there has been no examination of the preexisting nAbs present in the exposed infant against the exposure viruses—those circulating in the infected mothers. Examination of preexisting infant nAbs, immediately prior to the time of estimated transmission, against the maternal variants is most analogous to examining the efficacy of envisioned vaccine and passive immunization strategies.

Infants with higher nAb levels or with antibodies that mediate cellular cytotoxicity have also been shown to have lower mortality and morbidity (4–7). Previous studies, however, have not adequately examined how maternal anti-HIV-1 responses influence infant outcomes. Indeed, studies have suggested that HIV-1-exposed uninfected (HEU) infants have higher mortality than babies born to uninfected mothers (41). Association between maternal responses and infant outcome may provide insights into this important observation.

In this study, we examined nAbs present in 63 mother-infant pairs from a strict breastfeeding cohort in Malawi. The neutralizing capacity of infant and maternal plasma antibodies, present at the time of exposure, was evaluated against maternal strains and diverse heterologous viruses to assess responses against the exposure variants and neutralization breadth, respectively. We show that neutralization responses against the maternal strains do not differ among infants that acquired infection (AI) compared to HEU infants and TMs versus NTMs, and it does not associate with infant outcome. Maternal but not infant neutralization heterologous responses, however, were significantly associated with greater transmission risk and increased infant morbidity. Overall, this study has direct relevance to proposed vaccine efforts and ongoing passive immunization investigations as well as health outcomes for infants born to HIV-infected mothers.

## RESULTS

All maternal and infant plasma samples were obtained from the Breastfeeding, Antiretroviral, and Nutrition (BAN) Study (42) control arm in which the enrollees did not receive antiretroviral therapy after 7 days postdelivery. All samples from transmitting mother-infant pairs were obtained at an average of 41 days (range, 27 to 54 days) prior to the first infant sample with detectable HIV-1 plasma virus (see Table S1 in the supplemental material). For two infants (99 and 2315) and three mothers (99, 2315, and 1844), pretransmission plasma samples were only available 1 day postdelivery. For these cases, IgG was isolated from the infant and maternal sample to prevent interference from antiretrovirals in the plasma with subsequent analysis. Mother-infant pair 1844 was the only pair in which the date of sample collection differed between mother and infant (Table S1). Each of the 21 transmitting mother-infant pairs was matched to two nontransmitting mother-infant pairs by maternal plasma virus level, maternal absolute CD4^+^ T-cell count, maternal age, and duration of time after birth (Table 1).

10.1128/mBio.01373-17.3 TABLE S1  Clinical characteristics of transmitting and nontransmitting mother-infant pairs. Download TABLE S1, DOCX file, 0.1 MB. Copyright © 2017 Ghulam-Smith et al. 2017 Ghulam-Smith et al. This content is distributed under the terms of the Creative Commons Attribution 4.0 International license.

**TABLE 1:** Demographics for transmitting and nontransmitting mother-infant pairs^a^

Demographic parameter	Result for^b^:	
	TMs or AI infants	NTMs or HEU infants
Mothers	TMs	NTMs
Age, yr	26 (18–36)	25 (17–36)
Time PP, days	43 (1–256)	42.50 (12–297)
CD4 cells/mm^3^	336 (210–1,092)	339 (240–1,145)
Log_10_ plasma VL	4.78 (3.32–5.80)	4.67 (1.59–5.99)
Infants	AI infants	HEU infants
1st day detected as HIV^+^	83 (42–293)	
Birth wt, kg	2.95 (2.30–4.00)	3.05 (2.15–4.10)
% female	28.6	38.1
% with grade 4 SAE or death	38.1	14.3

^a^ TMs, transmitting mothers; NTMs, nontransmitting mothers; AI, acquired infection; HEU, HIV-exposed uninfected; PP, postpartum; SAE, serious adverse event; VL, virus level.

^b^ Median values are shown with ranges given in parentheses.

### Breadth and potency score reflects differences in neutralization capacity against global Env reference panel.

**FIG 1:**
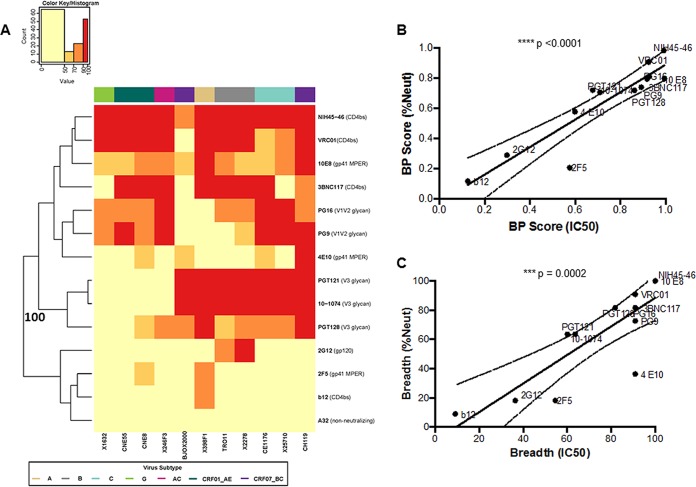
Breadth and potency are similar as assessed by the percentage of neutralization at the highest tested antibody concentration or IC_50_. (A) Each square in the heat map represents the average percentage of neutralization for each virus-MAb combination tested: <50%, yellow; 50 to 70%, light orange; 70 to 90%, dark orange; >90%, red. All MAbs were tested at a concentration of 50 µg/ml. Virus subtypes are indicated by color: A, khaki; B, gray; C, turquoise; G, lime green; AC, pink; CRF01_AE, dark green; and CRF07_BC, purple. The branches show the hierarchical clustering with bootstrap probability for 100 iterations. (B) Correlation between BP score estimated from the percentage of neutralization value at 50 µg/ml (*y* axis) and BP-IC_50_ (*x* axis). All IC_50_s are obtained from the Los Alamos database. (C) Correlation between breadth assessed using either the percentage of neutralization at 50 µg/ml (*y* axis) or the IC_50_ (*x* axis).

Neutralization responses were first assessed against a standardized reference panel consisting of 11 different Envs (43). These tests were done to assess heterologous responses, and this was not deemed reflective of activity against maternal strains. Neutralization against this reference panel was quantified using a breadth and potency (BP) score, which was estimated as the log-normalized average percentage of neutralization observed at one antibody concentration or plasma dilution across all 11 Envs (equation 1). Prior to examining neutralization capacity differences among the different plasma samples, BP score differences were assessed for diverse bnAbs. These controls were carried out to document that the BP score calculated using observed percentage of neutralization at one concentration adequately captures differences in neutralization capacity. BP scores were estimated for bnAbs directed at CD4bs (NIH-45-46, VRC01, 3BNC117, and B12), gp41 MPER (10E8, 4E10, and 2F5), V1V2 glycan (PG9 and PG16), V3 glycan (PGT128, PGT121, 10-1074), and gp120 (2G12) and a for a nonneutralizing antibody (A32). bnAbs with higher BP scores (NIH-45-46, VRC01, 3BNC117, 10E8, 4E10, PG9, PG16, PGT128, PGT121, and 10-1074) clustered separately with 100% bootstrap support from the other antibodies (Fig. 1A). bnAbs known to be more potent and with greater breadth (NIH-45-46, VRC01, 3BNC117, 10E8, PG9, PG16, PGT128, PGT121, and 10-1074) had significantly higher BP scores (median, 0.80; range, 0.70 to 0.99) than the other bnAbs (B12, 4E10, 2F5, and 2G12) (median, 0.25; range, 0.12 to 0.58; *P* = 0.001). Furthermore, BP scores for all bnAbs were higher than those for the nonneutralizing antibody, A32, which was essentially zero (BP score, 0.05), reflective of its minimal neutralization activity. A BP score calculated using the percentage of neutralization at one antibody concentration was also compared to a BP score calculated using the published 50% inhibitory concentration (IC_50_) value (BP-IC_50_) (equation 2). The BP-IC_50_ strongly correlated with the BP score estimated from using the observed percentage of neutralization at the highest tested antibody concentration (*P* < 0.0001, Spearman’s *r* = 0.94) (Fig. 1B). In addition, breadth assessed from the two different methods (the percentage of Envs neutralized >50% at the highest tested dilution and with an IC_50_ <25 µg/ml) were also highly correlated for the bnAbs (*P* = 0.0002, Spearman’s *r* = 0.87) (Fig. 1C). Thus, assessment of the potency and breadth derived from using the percentage of neutralization at the highest tested antibody concentration generated similar results to estimates derived from using serial dilutions to calculate an IC_50_. For all subsequent analyses against the reference Env panel, BP scores were estimated using the percentage of neutralization at the highest tested plasma concentration.

### Infected and HEU infants have similar neutralization responses against the heterologous Env panel.

**FIG 2:**
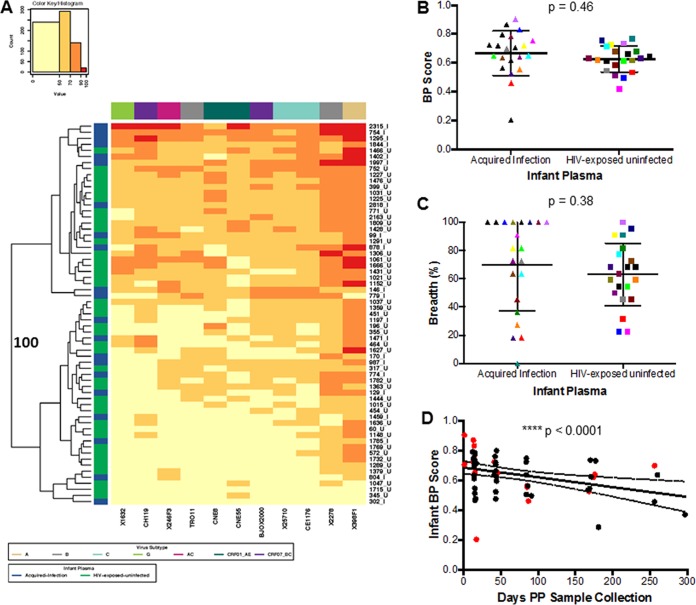
Infant neutralization response against a heterologous global Env panel. (A) Each square in the heat map represents the average percentage of neutralization for each virus-plasma combination tested: <50%, yellow; 50 to 70%, light orange; 70 to 90%, dark orange; >90%, red. Virus subtypes are indicated by color: A, khaki; B, gray; C, turquoise; G, lime green; AC, pink; CRF01_AE, dark green; and CRF07_BC, purple. On the left, blue denotes AI infants and green denotes HEU infants. The branches show the hierarchical clustering with bootstrap probability for 100 iterations. (B and C) Comparison of (B) breadth and potency score (BP) and (C) breadth alone between AI and HEU infants. Colors signify matched pairs. Each dot represents an average value from a minimum of 2 independent neutralization experiments for each infant. (D) Correlation between infant BP score and number of days after birth that samples were collected. The red and black dots indicate AI and HEU infants, respectively.

BP scores were compared among AI and HEU infant plasma samples at a time immediately prior to documented HIV-1 acquisition in the infected infant. Assessment against the standardized reference envelope panel was used to assess neutralization breadth and not responses against the exposure viruses. Heat map analysis showed that AI and HEU infants did not have unique neutralization fingerprints because the two infant clusters that separated with 100% bootstrap support contained relatively equivalent proportions of infected and uninfected infants (Fig. 2A). Interestingly, some of the highest BP scores were observed among the preinfection plasma samples (754, 1295, and 1844) and isolated IgG (2315) in four infants that eventually acquired infection (Fig. 2A). The AI group had a higher BP (median, 0.70; range, 0.20 to 0.90) than HEU infants (median, 0.63; range, 0.42 to 0.77), although this difference was not statistically significant (*P* = 0.46) (Fig. 2B). In addition, the AI infants (median, 81.82%; range, 0 to 100%) neutralized a higher percentage of the Envs in the heterologous panel compared to the HEU infants (median, 63.64; range, 22.73 to 100%), but this difference was not statistically significant (*P* = 0.38) (Fig. 2C). Among the AI group, 15 of the 21 infants neutralized more than half of the reference Env panel compared to 25 of 42 for the HEU group. In aggregate, there was no significant difference in preinfection plasma neutralization capacity among infants that acquired infection versus those that remained uninfected.

Infants receive the majority of passively acquired maternal antibodies in the third trimester (24). At birth, infant antibody levels often exceed those found in the mother (25), but maternal antibodies present in the infant plasma decrease over time even with breastfeeding (40). Infant BP scores showed an inverse correlation with the number of days between sample collection and birth (*P* = 0.0001, Spearman’s *r* = −0.50) (Fig. 2D). This suggests that HIV-exposed infants often possess HIV-1-specific maternal antibodies capable of broad and potent neutralization early after birth, but the presence of these antibodies wanes over time.

### The breadth and potency of maternal plasma antibodies significantly associate with vertical transmission and predetermined infant clinical outcomes.

**FIG 3:**
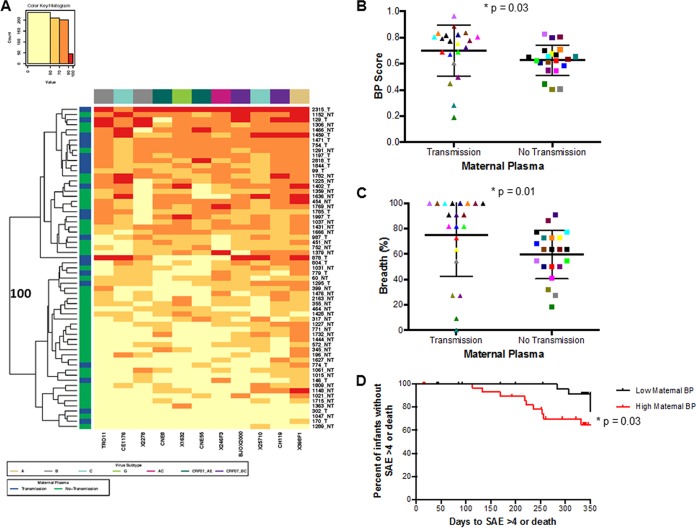
Maternal neutralization response against a heterologous global Env panel. (A) Each square in the grid represents the average percentage of neutralization for each virus-plasma combination tested: <50%, yellow; 50 to 70%, light orange; 70 to 90%, dark orange; >90%, red. Virus subtypes are indicated by color: A, khaki; B, gray; C, turquoise; G, lime green; AC, pink; CRF01_AE, dark green; and CRF07_BC, purple. On the left, blue denotes transmitting mothers (TMs) and green denotes nontransmitting mothers (NTMs). The branches show the hierarchical clustering with bootstrap probability for 100 iterations. (B and C) Comparison of (B) breadth and potency score (BP) and (C) breadth alone between TMs and NTMs. Colors signify matched pairs. Each dot represents an average value from a minimum of 2 independent neutralization experiments. (D) Kaplan-Meir curve estimating time (days) to a grade 4 SAE or death for infants born to mothers with BP greater than or equal to the cohort median (red) or less than the cohort median (black).

Infant plasma samples collected close to birth, when maternal antibody levels are high, showed the highest BP scores (Fig. 2D). Thus, BP was compared among TMs and NTMs to assess if maternal neutralizing capacity influences breast milk transmission. Maternal plasma samples were collected at the same time point before infection as infant plasma samples (with the exception of 1844) (Table S1). Heat map analysis showed that maternal samples clustered into two separate groups with 100% bootstrap support (Fig. 3A). These two clusters contained a significantly different proportion of TMs versus NTMs (*P* = 0.03; with Benjamini-Hochberg [BH] correction for multiple comparisons, *P* = 0.06) suggesting that transmitting compared to nontransmitting mothers contained a unique neutralization fingerprint. TMs (median, 0.77; range, 0.19 to 0.96) also had a significantly higher BP score than NTMs (0.64, range 0.40 to 0.83) (*P* = 0.03; BH, *P* = 0.09) (Fig. 3B). TMs (median percentage of viruses neutralized of >50%, 90.91%; range, 0 to 100%) also had significantly higher breadth compared to NTMs (median percentage of viruses neutralized of >50%, 63.64%; range, 18.18 to 90.91%) (*P* = 0.01; BH, *P* = 0.06) (Fig. 3C). The significantly higher BP observed in TMs was primarily driven by neutralization against specific isolates. TMs had a significantly higher response than NTMs against the subtype B, TRO11, subtype C, CE1176, and the recombinant CRF07_BC, BJOX2000 Envs (see Fig. S1 in the supplemental material).

10.1128/mBio.01373-17.1 FIG S1  Percentage of neutralization against individual Envs in the reference panel among transmitting and nontransmitting mothers. Virus and subtype are shown on the *x* axis. Red bars represent transmitting mothers (T); white bars represent nontransmitting mothers (NT). Statistically significant differences are labeled above individual Envs. Download FIG S1, TIF file, 0.1 MB. Copyright © 2017 Ghulam-Smith et al. 2017 Ghulam-Smith et al. This content is distributed under the terms of the Creative Commons Attribution 4.0 International license.

Previous studies found that passively acquired maternal antibodies may protect infants from adverse clinical outcomes (4, 5). A more recent study found an inverse association between passively acquired antibodies capable of antibody-dependent cellular cytotoxicity (ADCC) and infant mortality (6). Therefore, association between preinfection BP score and infant outcome was examined in our cohort. In this cohort, 14 infants had a grade 4 serious adverse event (SAE) or death during BAN Study follow-up. An SAE of grade 4 or death occurred in 8 and 6 AI and HEU infants, respectively (see Table S2 in the supplemental material). To examine the effect of BP scores on infant outcome, we compared infants and mothers with BP scores greater than or equal to the median cohort BP versus infants and mothers with BP scores less than the median cohort BP. Similar to the Milligan study (6), which also looked at neutralizing antibody activity using data from the Lynch study (40), we did not observe a significant association with infant BP score and infant outcomes (*P* = 0.79). However, there was a significant 3.4-fold increase in the likelihood of having a life-threatening illness or death in infants born to mothers with high BP compared to low BP (hazard ratio [HR], 3.39; 95% confidence interval [CI], 1.06 to 10.90; *P* = 0.03; BH, *P* = 0.06) (Fig. 3D). The risk was similar for infants who acquired infection compared to those who did not, although the results were not statistically significant, likely due in part to the small number of events (AI HR, 2.52, 95% CI, 0.37 to 16.80, *P* = 0.35; HEU HR, 2.40, 95% CI, 0.48 to 12.09, *P* = 0.29). Overall, these data demonstrate that infants born to mothers with a pretransmission broad and potent heterologous nAb response are more likely to both acquire HIV-1 and have a poor clinical outcome.

10.1128/mBio.01373-17.4 TABLE S2  Infant serious adverse events. Download TABLE S2, DOCX file, 0.1 MB. Copyright © 2017 Ghulam-Smith et al. 2017 Ghulam-Smith et al. This content is distributed under the terms of the Creative Commons Attribution 4.0 International license.

**FIG 4:**
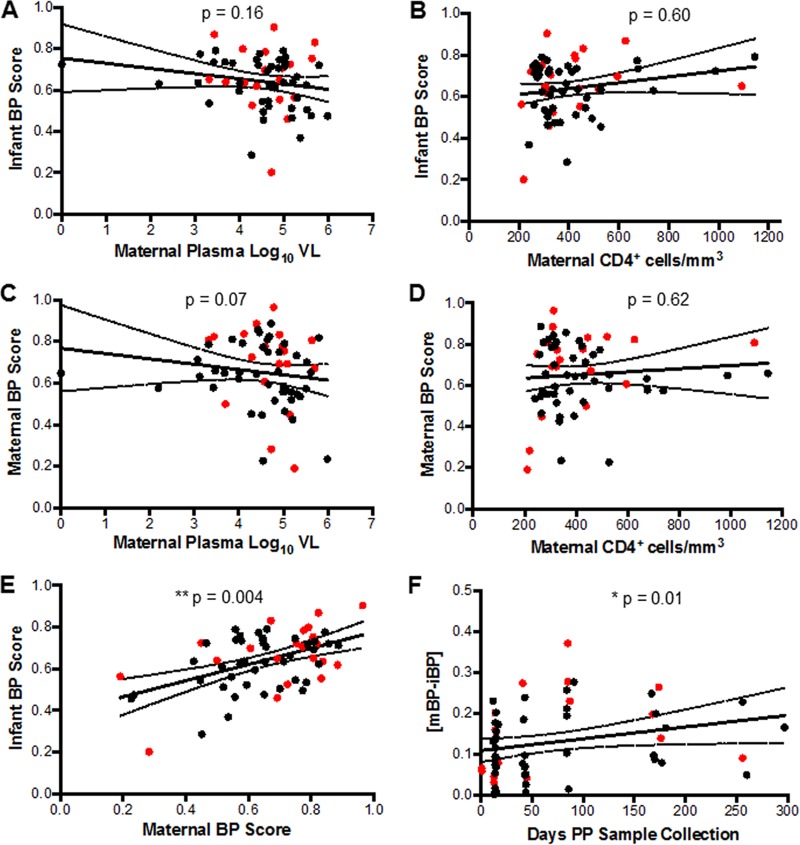
Infant and maternal BP score correlations. (A to F) Correlation between (A) infant BP score and maternal plasma virus level, (B) infant BP score and maternal CD4^+^ T-cell counts, (C) maternal BP scores and maternal plasma virus level, (D) maternal BP scores and maternal CD4^+^ T-cell counts, (E) infant and maternal BP scores, and (F) difference between maternal and infant BP scores and interval duration between birth and sample collection. The red dots indicate TMs and their AI infants. The black dots indicate NTMs and their HEU infants.

The emergence of heterologous neutralizing responses has been associated with a number of factors, including duration of infection and plasma virus level (44). Therefore, we assessed whether infant and maternal BP scores correlated with maternal clinical characteristics. Infant BP score did not correlate with maternal viral load (*P* = 0.16, Spearman’s *r* = −0.18) (Fig. 4A) or CD4^+^ T-cell count (*P* = 0.60, Spearman’s *r* = 0.07) (Fig. 4B). Maternal BP score, however, showed an inverse correlative trend with plasma virus level (*P* = 0.07, Spearman’s *r* = −0.23) (Fig. 4C), but there was no association with maternal CD4^+^ T-cell counts (*P* = 0.62, Spearman’s *r* = 0.06) (Fig. 4D). This suggests that maternal viral load and maternal advanced disease did not drive the association between maternal neutralization BP score and breast milk transmission.

Although maternal disease variables were not associated with preinfection infant BP scores, maternal and infant BP scores were highly correlated (*P* = 0.004, Spearman’s *r* = 0.36) (Fig. 4E). Furthermore, the correlation between maternal and infant BP scores was significantly associated with interval duration between birth and sample collection (*P* = 0.01, Spearman’s *r* = 0.32) (Fig. 4F). Maternal and infant samples obtained soon after birth had significantly lower BP difference compared to those obtained at later time points. Overall, this shows that infected mothers pass broad and potent HIV-1-specific nAbs to their infants especially close to birth and these nAbs are not protective against breast milk transmission.

### Passively acquired nAbs in breastfeeding infants do not protect against infection with maternal autologous virus.

HEU infants did not possess a significantly broader or more potent antibody response than those infants that acquired HIV-1 through breast milk. However, the heterologous viral isolates, although representative of the global population, are not the viruses infants need to neutralize in order to escape infection. During breast milk transmission, naive susceptible infants are only exposed to the quasispecies circulating in the chronically infected mother (31). Invariably, these maternal variants are expected to be dramatically different from the envelopes in the reference panel. We hypothesized that HEU compared to AI infants had a higher nAb response against their maternal variants. To examine this hypothesis, we isolated Envs from the maternal predocumented transmission plasma.

**FIG 5:**
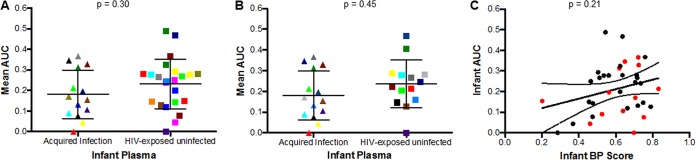
Neutralization response against maternal variants among infants that acquired infection and those that remained uninfected. (A and B) Area under the inhibition curve (AUC) values among AI versus HEU infants examined as (A) independent groups (Mann-Whitney test) and (B) matched pairs (linear regression model). Colors signify matched pairs for both analyses. (C) Correlation between infant AUC neutralization response against maternal strains and heterologous virus (BP score). The red and black dots indicate AI and HEU infants, respectively.

A recent study in lactating Malawian women found interspersion of plasma and breast milk HIV Env sequences, suggesting limited or no compartmentalization of breast milk variants (45). Furthermore, we have demonstrated that virus stocks incorporating either bulk PCR generated or a library of single-genome PCR-amplified Envs have similar neutralization susceptibility (46). Therefore, bulk PCR or a pool of envelopes amplified by single-genome amplification (SGA) were incorporated into an HIV-1 backbone to generate maternal virus stocks (see Table S3 in the supplemental material). Rather than testing neutralization against individual Envs, this nonselective methodology allowed assessment against quasispecies circulating in the infected mothers. Replication-competent viruses incorporating maternal Envs were successfully generated from 14 TMs and 23 NTMs. Among AI infants, 4 of 14 neutralized their mother’s virus above 50% at the highest tested plasma dilution (1:50) compared to 9 of 23 in the HEU group (*P* = 0.52). As many of the maternal and infant plasma samples did not yield 50% inhibition, the area under the inhibition curve (AUC) was calculated by taking the average neutralization within the range of dilutions tested for each antibody-virus combination (47). This yielded values ranging from 0 to 1, with 1 representing the most potent plasma neutralization response and 0 representing no neutralization (Table S3). There was a significant correlation between IC_50_ and corresponding AUC (*P* < 0.0001, Spearman’s *r* = 0.76) among the samples in which 50% or greater inhibition was observed at the highest tested dilution (see Fig. S2 in the supplemental material). This strong correlation suggests that AUC can be used as a correlate for IC_50_ and group comparisons using AUC can be done without excluding cases in which 50% inhibition could not be achieved. The median AUC in the AI group was 0.16 (range, 0.00 to 0.37), compared to 0.25 (range, 0.00 to 0.49) in the HEU group. Neutralizing activity against autologous maternal viruses was not significantly different when AI and HEU were examined as independent groups (*P* = 0.30) (Fig. 5A) or when comparing matched infant pairs (*n =* 14; *P* = 0.45) (Fig. 5B). In aggregate, HEU infants, compared to AI infants did not have greater neutralization capacity against their corresponding mother’s variants.

10.1128/mBio.01373-17.2 FIG S2  Inhibition area under the curve (AUC) and IC_50_ are highly correlated. Plots shows AUC (*y* axis) and IC_50_ (*x* axis) for different maternal plasma samples against autologous virus. Only plasma-virus pairs with an estimated IC_50_ of ≥50 are included in the graph. The text above shows the Spearman’s correlation coefficient and *P* value. Download FIG S2, TIF file, 0.1 MB. Copyright © 2017 Ghulam-Smith et al. 2017 Ghulam-Smith et al. This content is distributed under the terms of the Creative Commons Attribution 4.0 International license.

10.1128/mBio.01373-17.5 TABLE S3  Infant and maternal autologous IC_50_ and AUC with PCR method for maternal Env amplification. Download TABLE S3, DOCX file, 0.1 MB. Copyright © 2017 Ghulam-Smith et al. 2017 Ghulam-Smith et al. This content is distributed under the terms of the Creative Commons Attribution 4.0 International license.

While overall ability to neutralize their mothers’ virus did not differ among the HEU and AI groups, there were cases where infants had high neutralization capacity against maternal autologous virus but relatively limited neutralization responses against the global reference Env panel. Indeed, infant AUC against its maternal virus did not show a significant correlation with BP score (*P* = 0.21, Spearman’s *r* = 0.21) (Fig. 5C). This implies that, in infants, the neutralization response to heterologous virus is distinct from the neutralizing activity against the maternal exposure virus.

### Maternal neutralization against autologous virus does not predict transmission risk.

**FIG 6:**
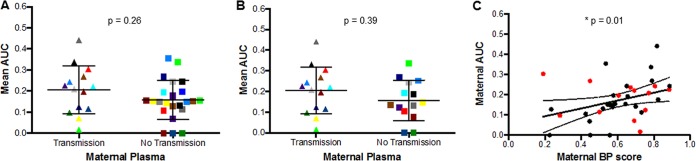
Autologous neutralization response among transmitting and nontransmitting mothers. (A and B) Autologous neutralization represented by (A) area under the neutralization curve (AUC) between TMs and NTMs examined as (A) independent groups (Mann-Whitney test) and (B) matched pairs (linear regression model). Colors signify matched pairs for both analyses. (C) Correlation between maternal neutralization response against autologous virus (AUC) and heterologous virus (BP score). The red and black dots indicate TMs and NTMs, respectively.

Over the course of infection, viruses evolve and the majority of circulating viruses are neutralization resistant to contemporaneous plasma (9). A higher proportion of TMs (5 of 14 [36%]) compared to NTMs (6 of 23 [26%]) neutralized their autologous virus above 50% at the highest tested plasma dilution, although this frequency difference was not statistically significant (*P* = 0.53). The TMs (median, 0.22; range, 0.02 to 0.44) had higher AUC against autologous virus than the NTMs (median, 0.15; range, 0.00 to 0.36), but this difference was also not statistically significant when examined as independent groups (*P* = 0.26) (Fig. 6A) or matched pairs (*n =* 14; *P* = 0.39) (Fig. 6B). In contrast to the infants, maternal AUC significantly correlated with their BP scores (*P* = 0.01, Spearman’s *r* = 0.42) (Fig. 6C). This concurs with a similar observation in a different cohort that also suggested that TMs have higher nAbs against heterologous and autologous viruses (48).

## DISCUSSION

In this study, we compared heterologous and autologous HIV-1-specific plasma antibody responses from subtype-C-infected TMs and NTMs and their respective infants. As participants in the control arm of the BAN Study (42), transmission mode was restricted to the breastfeeding period. Utilizing this unique cohort, we found that although HIV-1-specific nAbs were efficiently transferred from mother to infant; they were not a correlate of protection from HIV-1 breast milk acquisition. Indeed, we observed that some of the infants with the highest neutralization BP were infected through breastfeeding. Surprisingly, HIV-1-infected TMs compared to NTMs harbored greater pretransmission neutralization breadth and potency. Furthermore, infants born to mothers with higher pretransmission neutralization responses were more likely to have a serious adverse outcome. In summary, these data argue that in a natural HIV-1 transmission setting, presence of nAbs during exposure but prior to transmission does not protect against HIV-1 acquisition. Additionally, higher levels of anti-HIV-1 neutralizing responses in the mother associate with both transmission and infant morbidity.

Vaccine efforts or passive immunization protocols aim to have bnAbs present prior to HIV-1 exposure in susceptible individuals (49). Although animal models have demonstrated remarkable success in preventing infection when bnAbs are present prior to exposure (20, 21), it remains unclear if similar efficacy will be evident in natural human HIV-1 transmission (23). Examination of transmission frequency differences among individuals that harbor neutralization responses during exposure, similar to those envisioned by vaccine efforts and passive immunization protocols, is one way to partially explore this important question. Using a standardized panel of global Envs (43), we calculated a BP score to characterize the neutralization capacity in a plasma sample. Importantly, prior to plasma analysis, previously well-characterized bnAbs were used to validate that determination of neutralization at one concentration, as opposed to calculating an IC_50_, adequately captures differences in neutralization capacity (Fig. 1). AI infants compared to HEU had similar neutralization responses against the global Env panel (Fig. 2). This highlights that while some infants possessed antibodies capable of blocking diverse primary HIV-1 strains that have limited neutralization susceptibility, this combination of breadth and potency is not a correlate of protection. Our results obtained from a large number of mother-infant pairs in a strict breastfeeding subtype C transmission cohort confirm conclusions from a different study of mostly subtype A-infected individuals using a smaller, less diverse panel of viruses (40). In contrast to the prior investigation, in our study, samples were collected at a time point both close to and prior to infection instead of at birth. This is important because antibodies acquired by the infant from the mother decrease after birth even with breastfeeding (40). Because the global envelope panel does not adequately represent the maternal quasispecies, we also compared exposed infants’ ability to block infection from the circulating maternal viruses. To our knowledge, this is the first study that has examined the infant’s ability to block the exposure viruses. Thus, our investigation is the most direct test of preinfection neutralization capacity in restricting HIV-1 acquisition because we examined infant responses against circulating maternal strains during the narrow window of time prior to transmission.

We observed a number of infants that possessed antibodies with extensive breadth and potency prior to infection, and yet they nevertheless acquired the virus from their mother. There are a number of reasons these infants may have become infected even though they had neutralization responses analogous to those that may be provided by bnAbs. First, these infants likely became infected with maternal variants that were resistant to the transplacental and breast milk-acquired nAbs. Therefore, in contrast to animal models in which challenge viruses are invariably sensitive to the nAbs under investigation (23), human transmission can occur with variants that are resistant to the preinfection existing nAbs. Second, breast milk contains both cell-free virus and infected cells (50–52). We and others have previously demonstrated that even though nAbs potently inhibit cell-free virus, they may be less efficient in blocking cell-to-cell transfer (11, 53). Thus, unlike most animal models in which challenges consist of cell-free virions (23), human transmission may occur from an infected cell, and this mode of spread may be less susceptible to antibody inhibition. Third, we assessed neutralization capacity in infant plasma, yet nAb responses may be different at the site of virus acquisition, namely, the oral or gastrointestinal mucosa (54). Therefore, it is possible that passive immunization strategies alone may not yield adequate antibody levels at mucosal sites of invasion (55). Our studies suggest that breast milk HIV-1 transmission can occur even in the presence of potent and broad preinfection neutralization capacity in susceptible individuals.

While nAbs present in the infant at the time of infection do not protect against infection, this does not preclude the possibility that the presence of greater neutralization capacity in the infected mother limits transmission (33, 56). Contrary to this proposed protective role, however, we observed that TMs compared to NTMs had significantly higher neutralization BP (Fig. 3). Interestingly, other studies have also observed that TMs or transmitting mother-infant pairs have greater neutralization capacity than NTMs or nontransmitting mother-infant pairs (34–36, 38). In comparison to our study, these other investigations differ in many important ways, such as the number of individuals, use of either pre- or postinfection infant plasma, examination of *in utero* to postpartum MTCT, presence of different HIV-1 subtypes, and lack of relatively equivalent plasma virus levels and absolute CD4^+^ T cells among the transmitting versus the nontransmitting group. Even with these differences, surprisingly, these studies have yielded a consistent theme that greater neutralization capacity is associated with higher MTCT frequency. The biological reasons for this recurrent observation remain unclear. It is possible that a higher maternal HIV-1 antibody response may enhance transmission by forming HIV-1-IgG immune complexes that bind to the neonatal Fc receptor (FcRn) in the infant gastrointestinal tract or Fc-gamma receptors (FcγRs) on immune cells such as macrophages or dendritic cells. This attachment may facilitate subsequent viral migrations across mucosal surfaces (57–60). This antibody-dependent enhancement (ADE) hypothesis remains highly controversial primarily because there are *in vitro* studies that both support (61–63) and refute it (64). Interestingly, there is *in vivo* support for this idea because animals given antibody infusion compared to sham treatment were more likely to have virus present at distal tissues from the site of inoculation, suggesting that antibodies may enhance transport across mucosal barriers (65). Analogously, in a randomized trial HIV Ig infusion along with nevirapine (NVP) at the onset of labor was associated with a higher infant infection rate at birth compared to the group that received NVP alone (66). Although ADE was deemed unlikely to account for this curious observation, over subsequent follow-up, infection rates were the same in the two groups, potentially as HIV Ig concentrations decreased (66). Taken together, the observed reasons for the positive association between maternal neutralization capacity and transmission frequency during MTCT remain a recurring unexplained finding with potentially profound implications for passive immunization strategies.

In addition to a high BP score predicting infection, we also demonstrate an association between high BP and greater likelihood of the infant dying or suffering an SAE (Fig. 3D). This finding was not merely driven by the association of higher BPs among TMs because events were present both in the infected and uninfected infants (Table S2). HEU infants are known to have worse outcomes than infants born to HIV-uninfected mothers, although the reason remains unclear (67). Some postulate that exposure to circulating viral antigens is immunosuppressive in HEU infants (68, 69). An alternative hypothesis could be that high levels of broad and potent nAbs in mothers may reduce the transfer of IgGs against other potentially harmful pathogens, such as tetanus (70) and measles (71) or may alter the infant humoral response (72). On the other hand, infected infants that acquired HIV-1 from mothers with broad and potent nAb responses may have had poorer outcomes because they were infected with a greater variety of or more pathogenic variants. Neutralization breadth and potency have been associated with length of infection and HIV-1 genotypic diversity (44, 73). Mothers with high BP and a greater variety of circulating viruses may be more likely to transmit diverse or more pathogenic strains to their breastfeeding infants. Indeed, infection with multiple variants as opposed to a single variant has been associated with faster disease progression (74, 75). A high maternal neutralizing response prior to transmission has not been previously associated with unfavorable infant outcomes, and further studies in different cohorts are warranted.

Our results do confirm that infants acquire HIV-1-specific nAbs from their infected mothers. Maternal BP scores correlated with infant BP scores, and this correlation was strongest at the earliest time points after birth (Fig. 4). In addition, there was a significant correlation between sample time postbirth and infant BP (Fig. 2C). This implies that infants acquire maternal anti-HIV-1 specific nAbs after birth, and over time infants have *de novo-*generated non-HIV-1-directed antibodies, which dilutes the HIV-directed humoral component. The fact that the risk of breast milk transmission is greatest in the first 6 weeks’ postpartum (76), at a time when maternal antibody titers are at their highest, indirectly further suggests a lack of protection in the face of broad and potent HIV-1-specific nAbs.

Passively acquired preinfection nAbs against a heterologous Env panel did not distinguish infected versus HEU infants; however, neutralization of maternal variants, which are responsible for producing infection, is more meaningful in determining protection against exposure strains. AI compared to HEU infants had similar ability to neutralize their maternal exposure quasispecies (Fig. 5), but the majority of infants in both groups did not have a strong neutralizing response to their corresponding maternal virus as most did not reach 50% neutralization (Table S3). Remarkably, some infants with strong responses to heterologous viruses in the reference Env panel were unable to neutralize autologous maternal virus. This suggests that transmission occurs with a virus that may be resistant to a broadly neutralizing response, similar to what may exist with bnAb passive infusions (77). Most likely, however, maternal viruses are resistant to autologous antibodies acquired by the infant rather than harboring broad resistance against diverse bnAbs. Some infants that acquired HIV-1 demonstrated an ability to block the exposure quasispecies circulating in the infected mother. These infants may harbor antibodies that are capable of efficiently blocking maternal variants during cell-free but not cell-to-cell transfer. Future studies should compare the ability to block cell-to-cell spread among the preinfection plasma samples from infected and uninfected infants that had relatively high neutralization capacity against cell-free viruses with maternal Envs.

The TMs compared to NTMs had higher neutralization responses against autologous virus, but the difference was not statistically significant (Fig. 6). The same trend was also observed in a recent study comparing TMs and NTMs infected with mostly subtype A HIV-1 (48). In general, most mothers harbored quasispecies that were not susceptible to autologous antibodies (Table S3), confirming that most contemporaneous viruses escape neutralization responses (12, 13). Together, these data suggest that transmitting compared to nontransmitting mothers do not harbor more resistant variants that can escape neutralization and lead to transmission because NTMs and TMs harbor variants with similar susceptibility to autologous antibodies. Although the conservative BH adjustment yielded *P* values that suggest a statistical trend rather than significance, in every instance, greater maternal neutralization response was positively associated with an adverse outcome, which provides further support for our overall conclusions.

It is possible that antibody responses present in the breast milk are different from the ones in the blood. It is well known that breast milk contains a larger amount of IgA compared to blood, which primarily has IgG isotypes. A recent study found no significant difference in the ability of antibodies present in the breast milk to neutralize three different HIV variants among TMs and NTMs from the BAN cohort, although, breast milk HIV-specific IgA binding to a consensus HIV-1 Env gp140 was associated with a decreased risk of transmission (56). It has been hypothesized that secretory IgA (sIgA), which is enriched in breast milk, is blocking infection at the infant gastrointestinal mucosa (78, 79). HIV sIgA, however, is rarely detected in the saliva of HEU infants (80). Interestingly, Kuhn and others (81) detected sIgA significantly more often in the breast milk of TMs compared to NTMs, and IgA from infected individuals has been presumed to mediate enhancement of HIV infection *in vitro* (82). It is difficult to directly compare our finding to the previous sIgA studies, but there is no definitive evidence that preexisting maternal antibody response protects against breast milk HIV-1 transmission.

Using a natural infection model, our data suggest that preexisting antibodies with cross-reactive potential do not protect against subtype C breast milk transmission. An important consideration is that this study only assessed neutralization. Other antibody functionalities, such as ADCC, may play a role in providing protection, as was observed in the secondary analysis of RV144 vaccine trial in Thailand (83). Indeed, a recent MTCT study suggested a role of ADCC in protection from breast milk transmission in mothers with high viral load (84). Other investigations, however, found no association between ADDC present in the infant plasma or maternal breast milk and infant transmission risk (6, 56). Future studies that examine other antibody functionalities, especially against the exposure variants circulating in the infected mothers, are needed. These types of studies will provide insights for future vaccine efforts, passive immunization strategies, and factors associated with infant morbidity.

## MATERIALS AND METHODS

### Ethics statement.

The BAN Study was approved by the Malawi National Health Science Research Committee, the institutional review boards at the University of the North Carolina, the U.S. Centers for Disease Control and Prevention, and Boston University. All women provided written informed consent for themselves as well as on behalf of their infants. Buffy coats from anonymized uninfected donor volunteers were obtained from the Kraft Family Blood Donor Center at the Dana Farber Cancer Center after written informed consent.

### Study cohort.

Mother and infant plasma samples were acquired from the control group of the BAN Study. The BAN Study compared a maternal combination antiretroviral therapy (cART) regimen, infant nevirapine, or no therapy after the peripartum period in HIV-1 breast milk transmission frequency (ClinicalTrials.gov no. NCT00164736). All mother-infant pairs examined in this study were treated peripartum with single-dose oral nevirapine followed by zidovudine-lamivudine (ZDV-3TC) for 7 days postpartum. This brief peripartum treatment in the control arm was deemed ineffective to prevent subsequent breast milk HIV-1 transmission, and it was deemed likely to have an insignificant impact on the maternal Env quasispecies or antibody repertoire. An infant negative-DNA PCR at birth and 14 days postpartum was required for enrollment to rule out intrauterine and intrapartum transmission. Mothers were instructed to exclusively breastfeed for 24 weeks, and mother-infant pairs were followed and tested with sample collection at regular intervals for 48 weeks (42). An infant was deemed as having acquired infection through breast milk when HIV-1 RNA was detected in a follow-up plasma sample and the previously collected sample was negative. All infant samples examined in this study were obtained prior to the documented HIV-1 acquisition. Each infant that eventually acquired infection and the corresponding transmitting mother were matched to two mother-infant pairs with no documented transmission based on maternal plasma virus level, maternal CD4^+^ T-cell counts, and days postpartum to sample collection.

### Antibodies.

The following MAbs were obtained from the National Institutes of Health (NIH) AIDS Reagent Program: VRC01, NIH-45-46, 10E8, 3BNC117, PG9, PG16, PGT121, PGT128, 10-1074, 4E10, 2F5, 2G12, B12, and A32. For maternal and infant plasma samples collected within the first 7 days after birth (maternal, 99, 1844, and 2315; infant, 99 and 2315), IgG was isolated using the Melon gel IgG spin purification kit (Thermo Scientific, Pierce Biotechnology) according to the manufacturer’s instructions. The concentration of collected IgG was measured on the NanoDrop 2000 spectrophotometer (Thermo Scientific) and stored at 4°C for downstream use. An equivalent amount of isolated IgG to that present in a 1:50 plasma dilution was used in the subsequent neutralization assays.

### Cell cultures.

Human epithelial kidney HEK293T cells and TZM-bl cells were acquired from the NIH AIDS Reagent Program. Cells were maintained in Dulbecco’s modified Eagle medium (DMEM) with 10% fetal bovine serum (FBS) (Thermo Fisher Scientific), 2 mM l-glutamine, 100 U of penicillin per ml, and 100 μg of streptomycin per ml. Peripheral blood mononuclear cells (PBMCs) were purified using the Ficoll-Hypaque method from at least 3 separate HIV-seronegative donors and propagated in RPMI 1640 containing 10% FBS, 100 μg/ml penicillin-streptomycin, 5 μg/ml of phytohemagglutinin (Sigma), and interleukin-2 for 4 days prior to infection.

### Envelope isolation and amplification.

Expression plasmids for the 12 *rev-env* cassettes selected as global reference strains were acquired from the NIH AIDS Reagent Program. These 12 Envs were previously deemed to best represent the spectrum of neutralizing activity of a larger Env panel (43). Env amplicons were generated using PCR with primers IR delta ecto (5′-AAGCCTCCTACTATCATTAT) and envb3 out (5′-TTGCTACTTGTGATTGCTCCATGT) under previously described conditions (85). The QIAamp viral RNA isolation kit (Qiagen) was used to isolate RNA from maternal pretransmission plasma that was also used for the neutralization assessments. Envs were amplified using single-genome amplification (SGA) or bulk PCR under previously described conditions (86). All SGA-amplified amplicons or minimum of 3 independent bulk PCRs were pooled to generate a library of maternal Envs (Table S3).

### Replication-competent virus stocks.

Maternal Env pools and reference Envs were inserted into a subtype C T/F plasmid, pZM247Fv2 (87), and NL4-3 (AF324493) plasmid, respectively, using yeast gap repair homologous recombination with minor modifications from previously described methods (88, 89). Virus stocks were generated from HEK293T transfections as described previously (88). Briefly, HEK293T cells were cotransfected with a cytomegalovirus–NL4-3–long terminal repeat→Gag4 (CMV-NL4-3-LTR→Gag4) plasmid and a recombinant NL4-3 plasmid incorporating a reference Env or CMV-Q-23-LTR→Gag4 plasmid and plasmid pools incorporating maternal Envs. The supernatant was harvested 48 h posttransfection, filtered through a 0.45-μm-pore filter, and stored at −80°C. The 293T virus was passaged on PBMCs for a maximum of 7 days. At harvest, supernatants were centrifuged, filtered through a 0.45-μm-pore filter to remove cellular debris, and stored at −80°C. Titers of virus stocks were determined on TZM-bl cells in the presence of 10 μg/ml DEAE-dextran. A replication-competent virus could not be generated for reference panel subtype C Env CE0217, and thus the heterologous Env panel used in this study consisted of 11 variants.

### Neutralization assay.

All maternal and infant plasma samples were heat inactivated for 1 h at 56°C. All neutralization assays were performed in duplicate or triplicate a minimum of 2 independent times using TZM-bl cells as described previously (33). Briefly, neutralization of viruses incorporating maternal Envs was tested against 1:50 maternal or infant plasma and 2-fold serial dilutions. Neutralization of viruses incorporating a reference Env from the global panel was tested against either a 1:50 plasma dilution, an equivalent amount of isolated IgG to that present in a 1:50 plasma dilution, or 50 μg/ml MAb. A NL4-3 Env-deleted vesicular stomatitis virus G protein Env pseudotype was also used as a negative control in panel neutralizations. Virus was incubated with antibody, heat-inactivated plasma, or growth medium alone in a total volume of 50 μl for 1 h at 37°C, and approximately 1E5 TZM-bl cells with 10 μg/ml DEAE-dextran was added to each well after this incubation. After 48 h, infection levels were determined using Galacto-Light Plus (Applied Biosystems, Foster City, CA). Differences between relative light units (RLU) in the presence of antibody or plasma and growth medium alone were calculated as the percentage of neutralization. Background RLU in the TZM-bl cells alone were subtracted from all wells.

### Statistical analysis.

Neutralization responses against the global reference Env panel for each plasma sample and MAb were summarized by two different but related estimates. The first estimate, termed “breadth-potency” (BP), incorporated both the amount of neutralization against an Env at either a 1:50 plasma dilution or 50 µg/ml MAb concentration and responses against the entire 11 Env panel. BP was estimated by averaging the percentage of neutralization across all 11 Envs, and in this calculation, an Env-plasma/MAb combination that yielded a negative percentage of neutralization was assigned a value of 0. This mean was log_2_ transformed so the score ranged from 0 to 1, with 0 representing no neutralization and 1 being 100% neutralization against all Envs, as shown in equation 1:

(1) $$\text{BP}=\Sigma \log_{2}(\%\;\text{neutralization}/100+1)/11$$

The second estimate, termed “breadth,” was defined as the percentage of Env variants neutralized at >50% at the highest tested plasma/MAb concentration. As opposed to BP, breadth did not incorporate the observed degree of neutralization against an Env.

For autologous variants, an IC_50_ was calculated as the dilution that gave 50% inhibition. Cases were assigned an IC_50_ of 25 (half of the highest tested plasma dilution) when 50% inhibition was not observed. AUC was also estimated because IC_50_ could not be estimated for a large number of samples (47). Differences in frequencies among two groups were examined using a two-sample test of proportions. Differences among groups were analyzed using the Wilcoxon matched-pair test, and for these comparisons, average values were used for the 2 controls matched to each case. Linear regression models were also fit with generalized estimating equations (GEEs) because averaging estimates from the 2 matched controls for each case is not always deemed ideal. All measures were arcsine transformed for better fit in the GEE models. We considered nutrition supplementation, infant birth weight, maternal age, infant death, and serious adverse events (SAEs) in the infant as potential confounders of the relationship between AI and HEU infants and between TMs and NTMs and each of the scores of interest (BP, breadth, and AUC). Results were similar between the Wilcoxon matched-pair test and linear regression models; therefore, only *P* values from the latter are reported. Spearman’s rank tests were used to examine correlation among all continuous variables.

BP and breadth scores were also calculated for MAbs using published IC_50_s against the global reference Env panel variants. The IC_50_s of an antibody against a specific reference Env was obtained from the Los Alamos database (http://hiv.lanl.gov/catnap) (90). This MAb BP-IC_50_ score was generated using equation 2:

(2) $${\text{BP-IC}}_{50}=\Sigma \log_{2}(2-{\text{IC}}_{50}/\text{highest tested concentration})/11$$

In cases, where an IC_50_ could not be estimated because 50% neutralization could not be achieved, IC_50_ was set as the highest tested antibody concentration. The BP-IC_50_ approaches 1 for the potent antibodies that have low IC_50_ against the majority of virus variants. The BP-IC_50_ is 0 for MAbs that demonstrate no neutralization capacity. Breadth was defined as the percentage of reference Envs neutralized with an IC_50_ of <25 μg/ml.

Clinical adverse events for these infants were graded by the BAN Study investigators prior to our sample evaluations and according to toxicity tables from the Division of AIDS at the National Institute of Allergy and Infectious Diseases (NIAID) (42). We used Cox proportional hazard models to study the risk of grade 4 SAE or death as a function of BP score and adjusted for the matching in the data by using a robust estimate of the standard errors. For this analysis, BP scores were dichotomized as high (BP score ≥ cohort median) versus low (BP score < cohort median). Nutrition, maternal age, and infant birth weight were considered potential confounders in this model. We also considered an analysis stratified by HIV status of the infant.

The BH correction was used to control for possible inflation of the type I error rate due to multiple testing. The adjusted *P* value with BH correction is presented for instances of multiple comparisons where the unadjusted *P* value is <0.05.

### Heat map.

Heat maps were generated using the Los Alamos HIV sequence database heat map tool (https://www.hiv.lanl.gov/). All heat maps used hierarchical clustering with the Euclidean distance method. Bootstraps were generated using standard procedure in the Los Alamos tool.
