# Supplementary material for: Maternal but Not Infant Anti-HIV-1 Neutralizing Antibody Response Associates with Enhanced Transmission and Infant Morbidity
Source: mBio. 2017 Oct 24;8(5):e01373-17. doi: 10.1128/mBio.01373-17 (PMC5654929; doi:10.1128/mBio.01373-17)
Supplement: TABLE S1 [file mbo005173540st1.docx]

**Table S1. Clinical characteristics of transmitting and non-transmitting mother-infant pairs.**

| **Transmitting Mother-Infant Pairs** | | | | | **Non-Transmitting Control Mother-Infant Pairs** | | | | | | | |
| --- | --- | --- | --- | --- | --- | --- | --- | --- | --- | --- | --- | --- |
| **ID** | **Days PP Sample Collection** | **Days PP Infant 1st HIV^+^** | **Maternal CD4^+^ cells/mm^3^** | **Maternal log_10_ plasma VL** | **ID** | **Days PP Sample Collection** | **Maternal CD4^+^ cells/mm^3^** | **Maternal log_10_ plasma VL** | **ID** | **Days PP Sample Collection** | **Maternal CD4^+^ cells/mm^3^** | **Maternal log_10_ plasma VL** |
| ***99** | 1 | 55 | 326 | 4.97 | **1152** | 17 | 263 | 4.72 | **1627** | 14 | 324 | 4.81 |
| **129** | 174 | 240 | 309 | 4.39 | **1015** | 169 | 336 | 4.52 | **1782** | 171 | 293 | 4.68 |
| **146** | 41 | 83 | 266 | 5.14 | **1476** | 42 | 268 | 5.04 | **1289** | 43 | 342 | 5.99 |
| **170** | 85 | 127 | 210 | 5.24 | **1021** | 84 | 265 | 5.03 | **1363** | 84 | 334 | 5.20 |
| **302** | 17 | 45 | 219 | 4.73 | **1666** | 13 | 258 | 4.91 | **1061** | 12 | 291 | 4.99 |
| **754** | 13 | 54 | 626 | 3.44 | **1227** | 15 | 674 | 3.12 | **771** | 15 | 677 | 3.42 |
| **774** | 176 | 204 | 439 | 3.70 | **1715** | 181 | 392 | 4.28 | **1031** | 170 | 431 | 3.68 |
| **779** | 256 | 293 | 595 | 4.58 | **1047** | 256 | 528 | 4.55 | **317** | 260 | 527 | 5.00 |
| **804** | 87 | 129 | 322 | 5.08 | **1444** | 86 | 261 | 5.30 | **1379** | 84 | 315 | 5.53 |
| **878** | 13 | 56 | 248 | 5.02 | **1809** | 14 | 283 | 5.03 | **1431** | 14 | 317 | 5.02 |
| **987** | 45 | 90 | 306 | 4.91 | **1732** | 43 | 319 | 4.66 | **2163** | 43 | 281 | 4.89 |
| **1197** | 15 | 43 | 1092 | 3.32 | **752** | 12 | 1145 | 3.47 | **451** | 14 | 529 | 4.02 |
| **1295** | 14 | 42 | 457 | 5.70 | **572** | 15 | 428 | 5.18 | **60** | 16 | 364 | 5.62 |
| **1402** | 14 | 56 | 293 | 5.64 | **1148** | 15 | 314 | 5.18 | **345** | 297 | 240 | 5.37 |
| **1459** | 85 | 127 | 445 | 4.91 | **399** | 84 | 396 | 4.39 | **1769** | 91 | 493 | 4.54 |
| **1471** | 14 | 42 | 520 | 4.12 | **1428** | 14 | 988 | 1.59 | **464** | 14 | 738 | 2.19 |
| **1785** | 168 | 196 | 336 | 4.29 | **454** | 167 | 304 | 3.32 | **1225** | 177 | 322 | 3.85 |
| ***†1844** | 15 | 42 | 426 | 4.59 | **1306** | 14 | 360 | 4.43 | **1636** | 15 | 466 | 4.65 |
| **1997** | 44 | 85 | 423 | 4.08 | **1359** | 41 | 436 | 3.07 | **355** | 44 | 471 | 3.99 |
| ***2315** | 1 | 43 | 312 | 4.78 | **1466** | 15 | 312 | 4.46 | **1291** | 14 | 273 | 4.64 |
| **2818** | 43 | 85 | 412 | 5.80 | **1037** | 43 | 388 | 5.30 | **196** | 44 | 375 | 5.51 |

ID,identification number; PP, postpartum; VL, virus level.

*Denotes samples in which IgG was isolated from plasma.

†Indicates infant plasma sample collection time; maternal plasma was collected 1-day PP and IgG was isolated for subsequent analyses.
