# Supplementary figures and images for: Maternal but Not Infant Anti-HIV-1 Neutralizing Antibody Response Associates with Enhanced Transmission and Infant Morbidity
Source: mBio. 2017 Oct 24;8(5):e01373-17. doi: 10.1128/mBio.01373-17 (PMC5654929; doi:10.1128/mBio.01373-17)

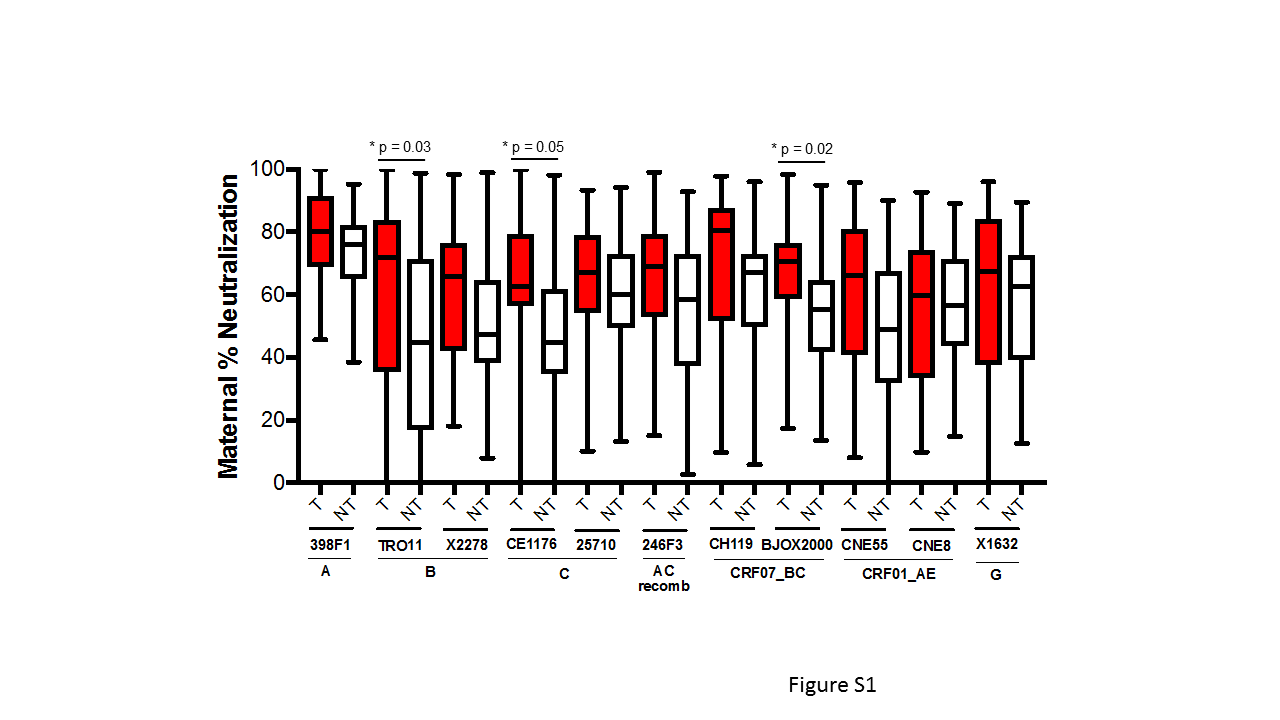

Supplement: FIG S1 [file mbo005173540sf1.tif]

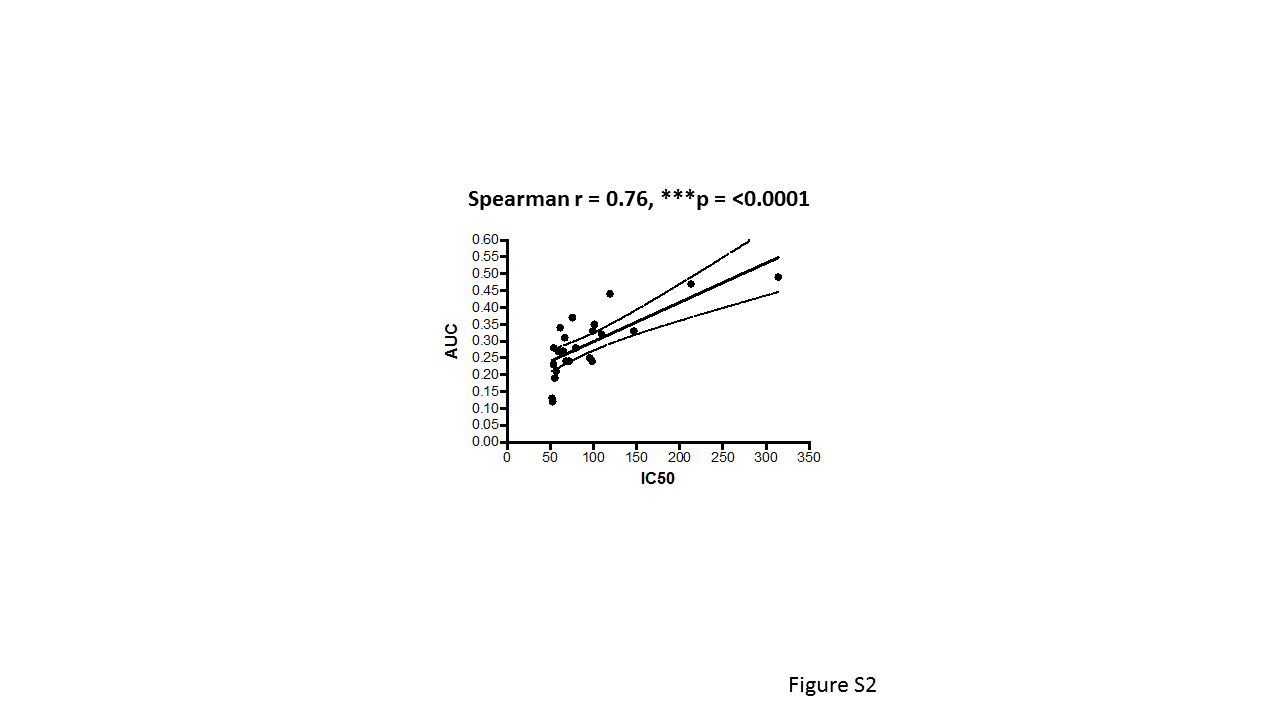

Supplement: FIG S2 [file mbo005173540sf2.tif]
