# Supplementary material for: Maternal but Not Infant Anti-HIV-1 Neutralizing Antibody Response Associates with Enhanced Transmission and Infant Morbidity
Source: mBio. 2017 Oct 24;8(5):e01373-17. doi: 10.1128/mBio.01373-17 (PMC5654929; doi:10.1128/mBio.01373-17)
Supplement: TABLE S2 [file mbo005173540st2.docx]

**Table S2. Infant serious adverse events.**

| **Patient ID** | **If infected, days PP to infant first HIV^+^ DNA PCR** | **Days to SAE or death** | **SAE type** | **SAE grade** |
| --- | --- | --- | --- | --- |
| 99 | 55 | 331 | Meningitis | 4 |
| 146 | 83 | 354 | Gastroenteritis | 5 |
| 170 | 127 | 306 | Gastroenteritis | 5 |
| 804 | 129 | 169 | Death | 5 |
| 878 | 56 | 221 | Death | 5 |
| 1459 | 127 | 253 | Pneumonia | 5 |
| 1471 | 42 | 256 | Death | 5 |
| 2818 | 85 | 235 | Pneumonia | 5 |
| 317 | -- | 351 | Anemia | 4 |
| 355 | -- | 352 | Anemia | 4 |
| 1225 | -- | 133 | Anemia | 4 |
| 1289 | -- | 283 | Death | 5 |
| 1379 | -- | 218 | Pneumonia | 4 |
| 1666 | -- | 113 | Death | 5 |

ID, identification; PP, postpartum; SAE, serious adverse event.
