# Supplementary material for: Maternal but Not Infant Anti-HIV-1 Neutralizing Antibody Response Associates with Enhanced Transmission and Infant Morbidity
Source: mBio. 2017 Oct 24;8(5):e01373-17. doi: 10.1128/mBio.01373-17 (PMC5654929; doi:10.1128/mBio.01373-17)
Supplement: TABLE S3 [file mbo005173540st3.docx]

**Table S3. Infant and maternal autologous IC_50_ and AUC with PCR method for maternal Env amplification.**

| **Patient ID** | **Transmission** | **PCR method** | **If SGA, # of amplicons combined** | **Infant Average IC_50_** | **Infant Average AUC** | **Maternal Average IC_50_** | **Maternal Average AUC** |
| --- | --- | --- | --- | --- | --- | --- | --- |
| 99 | TM | SGA | 12 | 25 | 0.17 | 56.64 | 0.21 |
| 1152 | NTM | SGA | 5 | 25 | 0.28 | 68.13 | 0.24 |
| 129 | TM | SGA | 18 | 66.63 | 0.31 | 53.66 | 0.23 |
| 1015 | NTM | SGA | 30 | 314.35 | 0.49 | 25 | 0.07 |
| 1782 | NTM | SGA | 29 | 109.38 | 0.32 | 25 | 0.18 |
| 146 | TM | SGA | 30 | 99.31 | 0.33 | 25 | 0.27 |
| 1476 | NTM | SGA | 12 | 25 | 0.37 | 25 | 0.14 |
| 1289 | NTM | SGA | 24 | 25 | 0.08 | 25 | 0.13 |
| 170 | TM | SGA | 12 | 25 | 0.20 | 25 | 0.30 |
| 1363 | NTM | SGA | 16 | 213.11 | 0.47 | 25 | 0.11 |
| 302 | TM | SGA | 5 | 25 | 0.15 | 25 | 0.10 |
| 1061 | NTM | BULK | -- | 25 | 0.13 | 25 | 0.00 |
| 771 | NTM | SGA | 10 | 25 | 0.24 | 25 | 0.20 |
| 774 | TM | SGA | 8 | 25 | 0.11 | 25 | 0.12 |
| 1715 | NTM | SGA | 17 | 25 | 0.00 | 25 | 0.00 |
| 779 | TM | SGA | 21 | 25 | 0.00 | 25 | 0.20 |
| 1047 | NTM | SGA | 30 | 25 | 0.15 | 25 | 0.00 |
| 317 | NTM | SGA | 9 | 25 | 0.28 | 25 | 0.16 |
| 804 | TM | SGA | 14 | 25 | 0.04 | 25 | 0.07 |
| 1444 | NTM | SGA | 16 | 53.74 | 0.28 | 25 | 0.15 |
| 1379 | NTM | SGA | 30 | 25 | 0.29 | 25 | 0.14 |
| 878 | TM | SGA | 8 | 75.61 | 0.37 | 25 | 0.12 |
| 1431 | NTM | BULK | -- | 61.21 | 0.27 | 64.87 | 0.27 |
| 987 | TM | BULK | -- | 101.05 | 0.35 | 25 | 0.22 |
| 2163 | NTM | BULK | -- | 25 | 0.25 | 55.04 | 0.19 |
| 1295 | TM | BULK | -- | 25 | 0.21 | 146.72 | 0.33 |
| 572 | NTM | BULK | -- | 59.27 | 0.27 | 52.09 | 0.13 |
| 60 | NTM | SGA | 10 | 25 | 0.15 | 71.73 | 0.24 |
| 1402 | TM | BULK | -- | 25 | 0.08 | 98.52 | 0.24 |
| 1148 | NTM | BULK | -- | 95.62 | 0.25 | 25 | 0.15 |
| 345 | NTM | SGA | 18 | 25 | 0.04 | 25 | 0.35 |
| 399 | NTM | SGA | 16 | 25 | 0.15 | 25 | 0.15 |
| 1785 | TM | BULK | -- | 25 | 0.09 | 25 | 0.02 |
| 454 | NTM | BULK | -- | 79.40 | 0.28 | 61.25 | 0.34 |
| 2818 | TM | BULK | -- | 25 | 0.13 | 119.06 | 0.44 |
| 1037 | NTM | BULK | -- | 52.62 | 0.12 | 25 | 0.11 |
| 196 | NTM | BULK | -- | 25 | 0.20 | 25 | 0.15 |

| **Avg IC_50_** | 25 | 50-70 | 70-100 | >100 |
| --- | --- | --- | --- | --- |
| **Avg AUC** | 0.00-0.20 | 0.20-0.30 | 0.30-0.40 | >0.40 |
|  |  |  |  |  |

ID, identification; TM, transmitting mother; NTM, non-transmitting mother; SGA, single genome amplification
